# Linking Structural and Mechanical Properties to Multimodal Sensory Perception of Fibrousness in Plant‐Based Meat Analogs

**DOI:** 10.1111/jtxs.70111

**Published:** 2026-08-20

**Authors:** Christina J. Birke Rune, Davide Giacalone, Mathias P. Clausen, Miek Schlangen

**Affiliations:** ^1^ Department of Green Technology University of Southern Denmark Odense Denmark; ^2^ SDU Climate Cluster University of Southern Denmark Odense Denmark

**Keywords:** computed fiber score/Fiberlyzer, fibrousness, image analysis, plant‐based meat alternatives, sensory–instrumental relationships, texture perception

## Abstract

Texture, particularly fibrousness, is a critical driver of consumer acceptance of plant‐based whole‐cut meat analogues (PBMAs), as it underpins the characteristic bite and tearing behavior associated with meat. Despite advances in producing and instrumentally characterizing fibrous structures in PBMAs, it remains unclear how macrostructural fiber organization and mechanical anisotropy translate into the human perception of fibrousness during eating, and how visual cues influence this perception. To address this, five PBMA samples with systematically varied fibrous structures were evaluated using a combined structural, mechanical, and sensory approach. Macrostructural fibrousness was quantified using the image‐based Fiberlyzer method, while directional compression testing characterized mechanical anisotropy and fluid release behavior. A trained sensory panel (*n* = 8) assessed visible and oral *fibrousness* together with related texture attributes under both visual and blindfolded conditions in order to determine the influence of visual appearance on texture perception. Results showed strong correspondence between image‐derived fiber structure and perceived *fibrousness*. The Fiberlyzer Fiber Score explained 84% of the variance in visible fibrousness and more than 85% of the variance in oral fibrousness during mastication (*p* < 0.05). Multivariate analyses further demonstrated that sensory *fibrousness* clustered with macrostructural fiber alignment and fracture‐related mechanical properties, while *juiciness* was associated with differences in compression‐induced fluid release. Removing visual cues increased response variability but did not significantly alter *fibrousness* perception for most samples, indicating that oral perception of *fibrousness* is primarily driven by physical structure rather than visual appearance. Together, these results demonstrate that macrostructural fiber organization quantified from images provides a reliable predictor of perceived *fibrousness* and that combining image‐based structural analysis, directional mechanical testing, and sensory evaluation offers a robust framework for interpreting fibrous texture in plant‐based meat analogues.

## Graphical abstract

Macrostructural fiber alignment quantified by the image‐based Fiberlyzer method strongly predicts both perceived visible and oral fibrousness in plant‐based whole‐cut meat analogs. Integrating structural imaging, directional mechanics, and sensory analysis provides a robust framework to interpret and design fibrous texture independent of visual appearance.

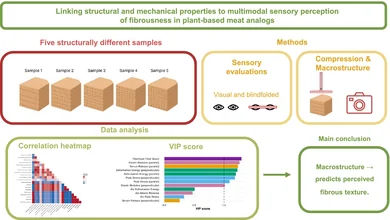

## 1 Introduction

Growing interest in plant‐based meat analogues (PBMAs) is driven by sustainability, health, and ethical considerations. Despite the rapid expansion of the product category, however, consumer acceptance remains uneven. Sensory quality, particularly texture, is consistently identified as a major limiting factor, with many PBMAs failing to meet expectations established by animal meat (Giacalone et al. 2022; Michel et al. 2021). Consumer sensory studies further indicate that meat products are consistently preferred over PBMAs on textural attributes, including fibrous or stringy structures, which contribute strongly to overall liking (Collier et al. 2025; Knaapila et al. 2024). From an instrumental structural and mechanical perspective, this shortcoming reflects the difficulty of reproducing the characteristic bite of meat, which arises from its anisotropic, hierarchically fibrous muscle structure (Schreuders et al. 2021). Recent work has demonstrated that instrumental structural and mechanical anisotropy in PBMAs strongly correlates with the formation of meat‐like fibrous structures (Wilhelm et al. 2025; Zink et al. 2024).

From a sensory perspective, texture is multidimensional, encompassing mechanical, geometrical, and surface characteristics (Bourne 2002). Fibrousness is a geometrical texture attribute that reflects both the spatial organization of the protein matrix and its fracture behavior during mastication. In the context of this study, fibrousness is defined as the perception of a layered or strand‐like texture during chewing and the extent to which the product breaks into fibers or strands. At low fibrousness, materials resemble isotropic gels, whereas pronounced fibrousness results in anisotropy, with structures that align and tear preferentially in specific directions, comparable to muscle tissue (Dekkers et al. 2018; Zink et al. 2024). Thermomechanical processing methods such as high‐moisture extrusion and shear cell technology can induce such anisotropic structures from plant proteins, although the resulting fibrousness depends strongly on both formulation and process parameters (Cornet et al. 2022; Dekkers et al. 2018; Schlangen et al. 2025; Zhang et al. 2022). Although fibrousness is rooted in structural anisotropy, it is not directly measurable and is therefore commonly assessed using instrumental proxies (Schreuders et al. 2021), even though instrumental measurements quantify material behavior rather than sensory texture itself, which emerges through perceptual integration during oral processing (Chen 2009).

A variety of instrumental methods have been used to characterize fibrousness, including rheological, mechanical, and image‐based approaches (Dahl et al. 2025; Schlangen et al. 2025). Mechanical methods, including tensile testing, cutting tests, and compression tests, can measure anisotropy by comparing force‐deformation responses parallel and perpendicular to fiber alignment. Wilhelm et al. (2025) demonstrated that the degree of mechanical anisotropy was different between meat and PBMAs, which they explained by differences in fiber connectivity, meaning that meat possesses long, continuous fibers that are tightly bound by connective tissue, whereas PBMAs consist of more weakly linked fibers that separate more easily under stress. Novel approaches in rheology, such as measuring the Poynting effect, have also shown promise, with higher values observed in meat products compared to PBMAs (Giménez‐Ribes et al. 2024).

Complementing the physical measurements, the recently developed “Fiberlyzer” method was introduced as a standardized tool to quantify visible fibrousness from macroscale images (Ma et al. 2024). Fiberlyzer computes an objective Fiber Score by segmentation and shape analysis of macroscopically visible fibers that are often only assessed qualitatively. As a relatively new method, its broader applicability and correlation to other measurements of fibrousness, such as sensory attributes and instrumental mechanical methods, are of interest.

Despite a large body of literature on plant‐based meat analogues, explicit and well‐defined sensory evaluation of fibrousness remains limited. A structured literature search conducted as part of the present work confirmed that only a small number of studies have investigated fibrousness using sensory methods (see Appendix S1 for details). In total, fourteen studies (Auer et al. 2025; Barnés‐Calle et al. 2024; Ettinger et al. 2022; Farrokhi and Azizi 2025; Fu et al. 2023; Ge et al. 2025; Lee et al. 2024; Lu et al. 2025; Nisov et al. 2024; Richter et al. 2024; Rune et al. 2026; Shahbazi et al. 2022; Xue et al. 2025; Zhao et al. 2024) were identified that included sensory evaluation of fibrous texture in PBMAs, of which four employed consumer‐based approaches and ten used trained descriptive analysis. Among the descriptive analysis studies, only seven evaluated fibrousness as an oral texture attribute during mastication, and only six provided an explicit definition of the attribute. Across these studies, fibrousness is generally described in similar perceptual terms referring to the perception of elongated or strand‐like structures during chewing (Barnés‐Calle et al. 2024; Lee et al. 2024; Nisov et al. 2024; Rune et al. 2026; Shahbazi et al. 2022; Xue et al. 2025).

Importantly, only three of the identified studies have attempted to relate sensory fibrousness to instrumental measurements. Barnés‐Calle et al. (2024) reported associations between perceived fibrousness and instrumental shear‐ and puncture‐related texture parameters when comparing processing conditions, while Lee et al. (2024) observed relationships between sensory fibrousness and bulk mechanical resistance measured by texture profile analysis. Similarly, Shahbazi et al. (2022) linked fibrous sensory attributes to general firmness‐and shear‐based mechanical metrics. However, these studies relied on bulk mechanical descriptors and did not quantify macrostructural fiber alignment or directional mechanical anisotropy. Meanwhile, instrumental research has extensively characterized fiber alignment and anisotropic mechanical behavior in plant‐based meat analogues, but such structural descriptors have rarely been quantitatively linked to trained sensory evaluation of oral fibrousness. Consequently, the relationship between macrostructural fiber organization, instrumental mechanical behavior, and human perception of fibrousness during eating remains insufficiently established.

Against this backdrop, despite extensive instrumental characterization of fibrous PBMA structures (Schreuders et al. 2021; Zhang et al. 2023), it remains unclear whether controlled variations in apparent fibrousness result in systematic sensory differences and to what extent commonly used instrumental descriptors reflect perceived texture attributes (Ma et al. 2024; Wilhelm et al. 2025). Addressing these questions is essential for understanding how structural design choices impact sensory experience and for interpreting instrumental measures in a sensory‐relevant context, consistent with recent frameworks distinguishing material texture measurements from perceptually derived sensory texture (Chen 2026).

Visual information represents an additional, largely unexplored factor in this context. In sensory science, it is well established that appearance can bias judgments in other modalities, a phenomenon referred to as cross‐modal bias or halo effect (Lawless and Heymann 2010). In fibrous products, visible alignment of fibers provides salient visual cues of structure, which may create expectations of firmness or chewiness and potentially shape the way texture is judged during eating (Ma et al. 2024). While oral processing studies have shown that fibrous structure affects chewing patterns and muscle activity (Oppen et al. 2023), little is known about how visual access modifies the perception of fibrousness and related attributes in PBMAs.

The present study was designed to fill these gaps by investigating the relationships between structural, mechanical, and sensory properties in plant‐based meat analogues. Specifically, we studied:

- How controlled variation in fibrousness translates into differences in sensory perception of plant‐based meat analogues.
- How sensory evaluations differ when assessors have visual access to the product compared to when vision is excluded (i.e., perception of fibrousness based on oral processing alone).
- To what extent instrumental measurements of computed Fiber Score (Fiberlyzer) and mechanical anisotropy (compression testing) linearly predict perceived sensory attributes.

Five samples—produced using by systematically varying processing conditions to generate products with different apparent fibrous structures—were evaluated by descriptive sensory analysis, complemented with compression testing and image‐based macrostructural characterization using the Fiberlyzer method. This study thus provides new insights into how variations in fibrous structure are translated into sensory perception, and how structural cues and cross‐modal influences shape the perception of fibrousness in PBMAs. Additionally, the study builds on and extends the original Fiberlyzer study (Ma et al. 2024) by integrating Fiber scores with mechanical and sensory data.

## 2 Materials and Methods

### 2.1 Samples and Preparation

Five plant‐based meat analogue products with varying degrees of visual and mechanical fibrousness were obtained from Plant Meat Makers B.V. (Amersfoort, the Netherlands). The products were formulated with soy protein and wheat gluten as primary ingredients (Ingredients: water, 26% texturized plant protein (gluten, soy), natural aromas, herbs and spices, salt) and manufactured at five different processing conditions using a variation of Couette cell technology (Krintiras et al. 2015, 2016). Products were frozen until use. Frozen products were thawed in the fridge (4°C) for 24 h prior to preparation or analysis. Thawed products were cut into cubic samples ensuring orientation of fibers was always marked (30 × 30 × 30 mm, roughly 40 g) and sous vide cooked in a water bath at 60°C for 30 min. The cooking protocol was applied consistently to all samples used for sensory evaluation, instrumental analyses, and image acquisition throughout the study. This cooking method was selected to ensure consistent thermal processing across all samples and facilitate logistics regarding sensory evaluation.

### 2.2 Macrostructure Image Acquisition and Visible Fibrousness Quantification

The macrostructure of the products was quantified using Fiberlyzer, an automated image analysis method to characterize visible fibrousness (Ma et al. 2024). The products were thawed before cutting specimens of 30 × 30 × 30mm cubes. To expose the internal fibrous structure, specimens were manually bent and secured using pins. Specimen were positioned in a lightbox equipped with an Aputure light source operated at 100% intensity. Images were captured in RAW format using an Iphone 16 Pro. A minimum of 6 images was acquired per sample. Captured images were manually cropped to a region of interest. Three representative photographs per sample used for Fiberlyzer analysis are provided in Appendix S2. Fiber Scores were computed using Fiberlyzer with a threshold of 72% and a minimum area of 0.01%. The threshold and minimum area parameters were previously calibrated against sensory panel assessment using a grid search (see Appendix S3).

### 2.3 Mechanical Properties in Compression

The mechanical properties of the products were analyzed using compression tests with a texture analyzer (TA.XTPlusC, Stable Microsystems, Surry, United Kingdom) equipped with a 30 kg load cell and a cylindrical probe (diameter 75 mm). Compression testing was selected as the most appropriate method given the product geometry. Cylindrical specimens (18 mm diameter, 28.5 mm height) were cut from the products using a core borer in both parallel and perpendicular (compressed from the top, along the fiber axis, and side, across the fiber axis) orientation to the fiber direction. Cylindrical specimens were preferred over cubic specimens to ensure uniform contact with the compression probe, avoid edge artifacts, and facilitate comparison with literature. Prior to testing, each specimen was individually vacuum‐sealed in plastic bags and cooked sous‐vide at 60°C for 30 min to mimic the sample preparation of the sensory testing. Uniaxial compression tests were performed at a constant deformation rate of 2 mm/s until reaching 50% strain. A deformation rate of 2 mm/s was selected to characterize the fundamental mechanical behavior of the samples, rather than to replicate chewing conditions. Force and displacement data were recorded using the Exponent Connect Software (Stable Micro Systems, Surrey, United Kingdom) at a frequency of 200 Hz. The engineering stress *σ* (Pa) was calculated with Equation (1):

(1) $$\sigma \left(t\right)=\frac{F\left(t\right)}{A\left(t\right)}$$

where *F(t)* is the force and *A(t)* is the contact area between the specimen and the compression plate. We assume the specimen to be volume conservative, assigning it a Poisson's ratio of 0.5. The contact area at time (*t*) was calculated with Equation (2):

(2) $$A\left(t\right)=\frac{h_{0}}{h\left(t\right)}\times A_{0}$$

where $h_{0}$ is the initial height of the sample at *t* = 0, $h\left(t\right)$ is the height of the sample at time *t*, $A\left(t\right)$ is the contact area *A* at time *t*, and $A_{0}$ is the initial contact area of the specimen calculating with the measured diameter of the specimen.

The Engineering Strain ε (−) Was Calculated With Equation 3

(3) $$\varepsilon \left(t\right)=\frac{h\left(t\right)}{h_{0}}$$

where *h*_*0*_ is the initial height of the specimen and *h(t)* is the height at time *t*. The Peak Stress was defined as the maximum stress achieved during the compression test. Deformation Energy was quantified as the area under the stress–strain curve, calculated using Simpson's rule. This parameter is also referred to as *toughness* in mechanics. In the present study, the term *deformation energy* is used to emphasize its interpretation as the mechanical work associated with compression (Peleg 2019). Elastic modulus (apparent Young's modulus) was determined by finding the longest initial linear region with *R* ^2^ > 0.998 after excluding the first 50 data points to eliminate noise, then calculating the slope of this linear section. Serum Release during compression was analyzed using the method from (Rudge et al. 2025). *Serum Release* refers to the fluid released from the food matrix during mechanical compression, which may contain water, fat, proteins, and solid particles (Y. Zhang et al. 2026). Whatman Grade 4 filter paper was placed above and below each specimen during compression testing, with serum release calculated as the difference in filter paper weight before and after compression. For each mechanical parameter, anisotropy indices (AIx) were calculated as the ratio between values measured parallel and perpendicular to the fiber direction. For each product, five specimens were analyzed parallel and five specimens were analyzed perpendicular to the fiber direction (*n* = 5 per orientation).

### 2.4 Sensory Descriptive Analysis

#### 2.4.1 Panel Training and Sample Evaluation

Eight trained assessors (six males, two females, mean age = 26.6 years old) participated in this study. All assessors had previously passed a sensory screening test in accordance with ISO 8586 (2012) and completed a comprehensive 20‐h training program in sensory evaluation of food texture in general, and more specifically in different plant‐based meat products. This training covered general sensory science theory, food texture theory, including mechanical, geometrical, body, and surface characteristics, using various food items as texture references, and standardized chewing techniques (Bondu et al. 2022; ISO 5492 2009, 2020; Rosenthal and Chen 2023; Szczesniak 2002). In addition, all assessors had prior experience in a descriptive analysis study focused on the texture of plant‐based meat products (Rune et al. 2026).

For the current study, in addition to the comprehensive training, the panel underwent an additional three training sessions (2 h each; 6 h total) focused specifically on the five experimental samples for this present study. During these sessions, a sensory vocabulary was developed, and the panel was calibrated accordingly, with particular emphasis on evaluating the selected attributes at predefined chewing stages representing different phases of mastication. The final sensory vocabulary comprised seven texture and mouthfeel attributes. *Fibrousness* was evaluated at multiple stages of consumption, resulting in a total of nine evaluation points per sample. *Visible fibrousness* was assessed prior to oral evaluation. During oral processing, mechanical attributes included *hardness* (chew 1), *springiness* (chew 1), *cohesiveness* (chew 2), and *chewiness* (chew 10). Geometrical attributes included *fibrousness* (chews 5 and 10) and *denseness* (chew 1), and one body and surface attribute *juiciness* (chew 5). The selected evaluation points represented standardized oral‐processing stages and were chosen to enable comparison of samples at equivalent stages of mastication rather than to characterize the complete temporal evolution of sensory perception. Table 1 shows the attribute definition and anchor point specific words. The attribute intensity was evaluated on a 15 cm line scale, using the RedJade software, anchored from “a little” to “a lot”.

**TABLE 1:** Sensory vocabulary developed by the trained panel to describe the samples.

Attribute (chew)	Texture category, definition and chewing technique	Anchor points
VISUAL		
Visible fibrous (0)	Perception of layered or strand‐like texture felt during chewing. Does it look like it breaks into fibers or strands?	A little to a lot (no strands to very stringy)
ORAL		
First bite (1–2 chews)		
Hardness (1)	Mechanical texture attribute. Place the solid sample between the molar teeth and chew gently, evaluating the force required to compress the food.	A little to a lot
Springiness/Elasticity (1)	Mechanical texture attribute. Place the solid sample between the molar teeth and compress it partially Bounce‐ back after pressure.	A little to a lot (no bounce to very springy)
Denseness (1)	Geometrical texture attribute. Compactness after biting completely through it.	A little to a lot (spongy/airy to very compact)
Cohesiveness/	Mechanical texture attribute.	A little to a lot
Fracturability (2)	How well the sample holds together.	(slides apart to holds together)
Chewing phase (5 chews)		
Fibrous (5)	Geometrical texture attribute. Perception of layered or strand‐like texture felt during chewing. Does it feel like it breaks into fibers or strands?	A little to a lot (no strands to very stringy) References was tofu to a cooked chicken breast
Juiciness/Serum release (5)	Body and surface texture attribute. Perception of wetness or juiciness released during chewing.	A little vs. a lot
End phase (10 chews)		
Chewiness (10)	Mechanical texture attribute. Chew sample with molar teeth until phase change. Work required/time to masticate a solid product onto the state of swallowing.	A little to a lot
Fibrous (10) (perceived as layers or strands)	Geometrical texture attribute. Perception of layered or strand‐like texture felt during chewing. Does it feel like it breaks into fibers or strands?	A little to a lot (no strands to very stringy)

*Note:* Also mentioned are the numbers of chews at evaluation point, texture category (mechanical, geometrical, body and surface), definition, chewing technique, and anchor point wordings.

**FIGURE 1:**
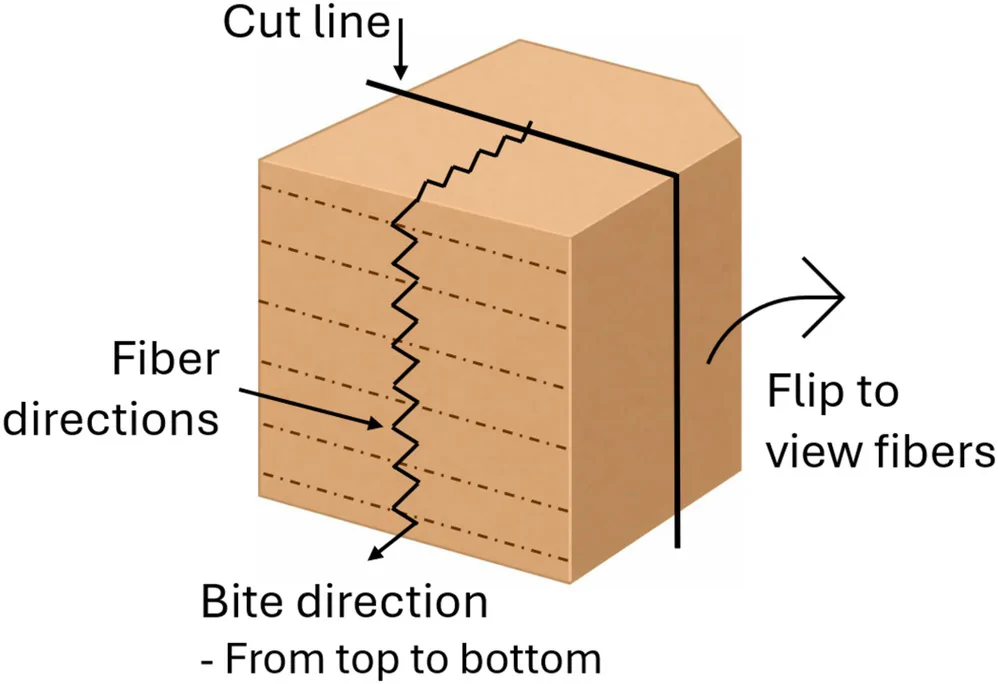
Figure showing how the sample was presented to the assessors on the plate. The top right corner was cut to ensure all samples were served with the fibers aligned in the same direction for all samples. Also is shown where the assessor was instructed where to cut and bite the sample. Dotted lines represent fiber direction.

The final assessment was conducted in a sensory lab, in individual sensory booths within a controlled laboratory environment (22°C; standardized booth lighting). Samples were served one at a time at five‐minute intervals in a randomized complete block design (Lawless and Heymann 2010). Each sample was presented on reusable white plastic (polypropylene) plates (Ø 20.5 cm), labeled with a random three‐digit code and evaluated at a serving temperature of 53°C (±2°C). The samples, cut into 30 × 30 × 30 mm cubes, were pre‐cut in one corner before cooking to ensure the fibers were aligned consistently across all samples and served with the precut in the top right corner (Figure 1). Assessors were instructed to cut (using a dark gray plastic (polypropylene) knife, 18 cm in length) the cube horizontally. Then the top half was flipped to the side making the fiber‐layers visible, and the visible fibrousness was evaluated. The remaining half was used to evaluate the oral attributes. The panel was instructed to bite into the center of the sample to ensure they bit down on the fiber‐layers, and that the panel evaluated an equal amount of sample each time across the panel. Assessors counted chewing cycles themselves and had received prior training and calibration in standardized chewing‐stage evaluation procedures before data collection.

A key component of the experimental design was the comparison between visual (consistent with standard procedures in descriptive sensory analysis) and blindfolded evaluations to assess the impact of visual input on sensory oral perception. To minimize memory and expectation biases, the two evaluation conditions were conducted on separate days, 5 days apart (Lawless and Heymann 2010; Stone et al. 2020). The order of presentation was counterbalanced across the panel: half of the assessors completed the blindfolded evaluation first and the visual evaluation second, while the other half completed them in reverse order. This counterbalancing was essential to control for potential day and order effects and to ensure that any observed differences could be confidently attributed to the evaluation condition itself (Lawless and Heymann 2010). All evaluations were conducted in triplicate within a single day per condition, following established protocols in descriptive sensory analysis where the focus is on identifying sample differences rather than assessing method reproducibility (ISO 3972 2011; Lawless and Heymann 2010; Stone et al. 2020).

During the blindfolded condition, assessors did not evaluate visible fibrousness. Samples were pre‐cut by the sensory staff prior to serving and served in a standardized manner to minimize non‐visual cues (Lawless and Heymann 2010), in line with general recommendations for controlling test conditions in sensory evaluation (ISO 8589 2010). After biting the sample, the remaining portion was discarded into a spittoon, and blindfolds were removed prior to completing the evaluation on a computer. To clarify, the assessor never saw nor touched the samples; only the sensory staff handled the samples in the blindfolded condition.

Each session began with warm‐up samples representative of the test set to help assessors recalibrate before formal evaluation (Lawless and Heymann 2010). Assessors were given a 10‐min break after the warm‐up and between each replicate to minimize sensory fatigue and maintain evaluation quality (Stone et al. 2020; Szczesniak 2002). Sparkling water (room temperature) and cold, green, seedless grapes were provided throughout the sessions as palate cleansers, approaches widely recommended for reducing residual sensory stimuli and restoring oral freshness (Lawless and Heymann 2010; Stone et al. 2020).

### 2.5 Data Analysis

All analyses were conducted in R version 4.3.3 (R Core Team 2024). For analyses of inferential nature, a 5% alpha level was considered to indicate statistically significant effects.

#### 2.5.1 Descriptive Analysis

Descriptive sensory data were analyzed using linear mixed‐effects models with Sample and Condition (blind vs. visual) as fixed effects, and Assessor, Replicate, and Session as random effects (lme4; Bates et al. 2015; Kuznetsova et al. 2017). Significant differences between samples were identified using Tukey's HSD post hoc tests (multcomp) with compact letter displays for interpretation.

The attribute *V_Fibrous* was only collected in the visual condition and was therefore excluded from blind and cross‐modal models. Panel agreement was evaluated with Kendall's W (irr; Gamer et al. 2012), and design effects of replicate, serving order, and session were tested and confirmed to be negligible.

#### 2.5.2 Structure–Serum–Sensory Relationship Analysis

Instrumental mechanical texture data, serum release, Fiberlyzer score, and sensory DA data were integrated at the sample‐means level for multivariate structure–sensory relationship assessment. Given the limited number of experimental samples, multivariate analyses were used primarily as exploratory tools to identify associations between structural, mechanical, and sensory domains rather than to develop predictive models. All variables were inspected and cleaned prior to analysis, with sample identifiers set as row labels and extraneous characters removed from variable names. Prior to correlation and multivariate analyses, all instrumental, structural, serum‐release, and sensory variables were mean‐centered and scaled to unit variance. This preprocessing was applied because the analyses integrated variables measured in different units and numerical ranges, thereby preventing variables with larger numerical magnitudes from disproportionately influencing the multivariate results.

Relationships between instrumental, serum‐related, structural, and sensory variables were screened using pairwise Spearman rank correlations with pairwise deletion of missing data.

Partial Least Squares Regression (PLSR) was used to explore multivariate structural predictors of sensory texture (using the package pls; (Mevik and Wehrens 2007)). Fiberlyzer score, instrumental Peak Stress, Deformation Energy, Elastic modulus, and Serum Release metrics were entered as X variables, while sensory texture attributes served as multivariate Y responses. Leave‐one‐out (LOO) cross‐validation was performed at the sample level, whereby one observation (row) was removed at a time, the model was refitted on the remaining data, and the excluded sample was predicted. This procedure was applied iteratively across all samples to mitigate overfitting and assess model robustness. Explained variance per component was extracted to support model interpretation. Predictor loading weights and sensory loadings were visualized in a biplot to aid interpretation of shared variance and grouping of predictor domains (ggrepel; Slowikowski 2025). Predictor variables were grouped post hoc (e.g., “Peak Stress”, “Deformation Energy”, “Serum Release”) to facilitate structural interpretation.

Variable Importance in Projection (VIP) scores were computed from the fitted PLSR model to quantify the relative contribution of each instrumental predictor to sensory variance. VIP values were derived from the PLS predictor weight matrix, component scores, and sensory loadings, weighted by the amount of Y‐variance explained by each PLS component. Predictors with VIP ≥ 1.0 were considered influential drivers of sensory texture (Wold et al. 2001).

Associations between Fiberlyzer score and sensory fibrousness attributes (Visible Fibrous, Fibrous 5 and 10 chews) were tested using simple linear regression models, with model *R* ^2^ and significance levels reported. Scatterplots with regression lines and 95% confidence bands were generated for visual interpretation of effect size and direction. To further validate findings, regressions were replicated in a subset where fibrousness was evaluated under both visual and blind test conditions.

## 3 Results

### 3.1 Fiber Score at Macroscale

**FIGURE 2:**
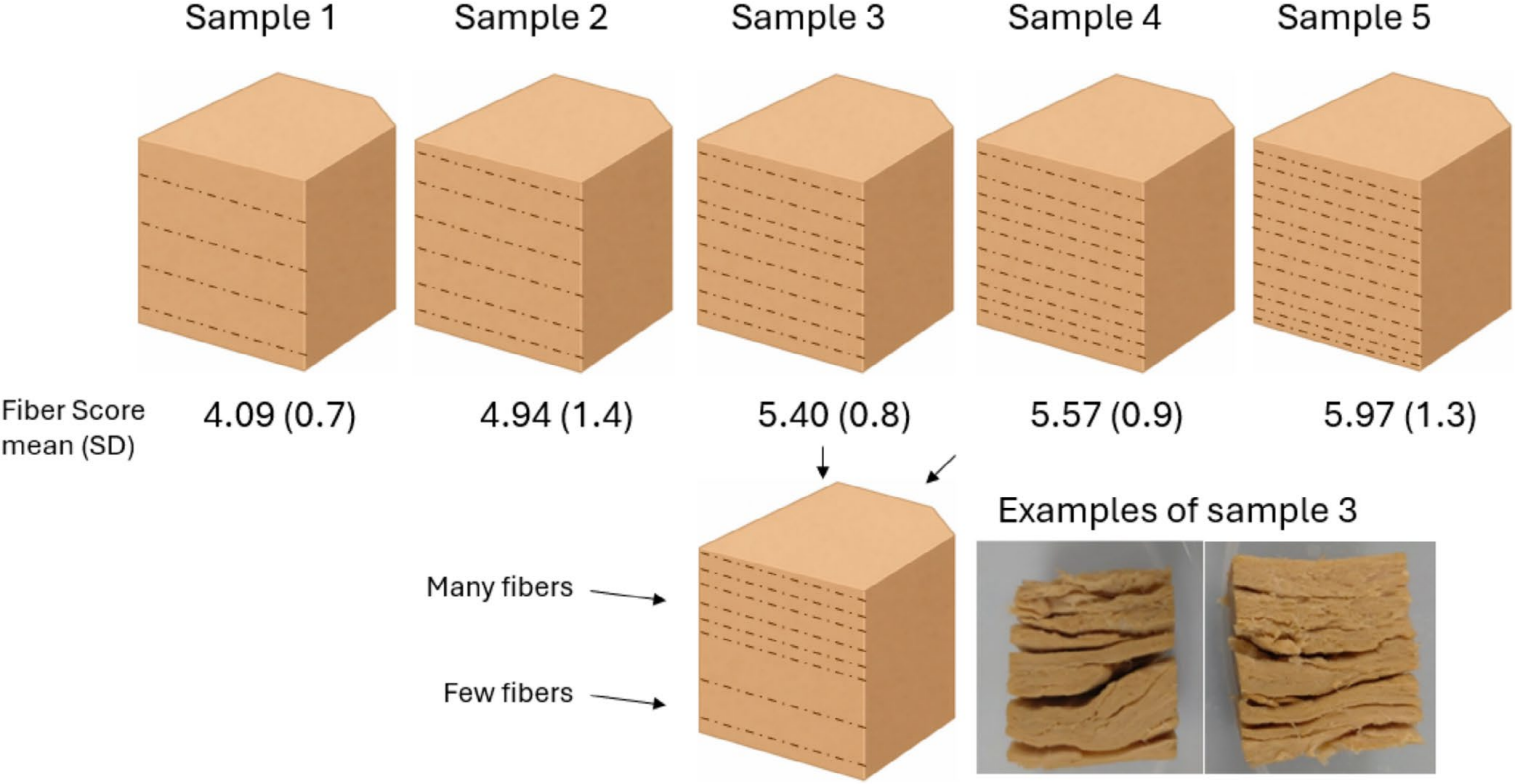
Illustrations of the five samples and their increasing fibrous structure and their corresponding Fiberlyzer Fiber Scores (mean ± SD). The illustrations are schematic representations intended to visualize the overall structural differences among samples and were not used for Fiberlyzer analysis. Fiber Scores were calculated from a minimum of six replicate photographs per sample as described in Section 2.2. Due to within‐sample structural variation, the illustrations are intended to represent the overall progression in fibrousness rather than individual sample photographs. An example photograph of Sample 3 separated into two layers is included to illustrate the layered and strand‐like structural features associated with fibrousness in the products.

The Fiber Score at macroscale obtained through Fiberlyzer analysis revealed clear differences in visible fibrousness across the five samples. Mean Fiber Scores increased progressively across samples, from Sample 1 (4.09 ± 0.68) < Sample 2 (4.94 ± 1.38) < Sample 3 (5.40 ± 0.78) < Sample 4 (5.57 ± 0.92) < Sample 5 (5.97 ± 1.34), indicating a broad range of structural alignment within the sample set (see Figure 2).

A degree of natural structural variability was present across all samples. Fibrous structure was observed in every sample and was particularly noticeable in the middle samples, most prominently in Sample 3. In some samples, two distinct fiber layers were present within a single sample: one comprising thicker, platelike strands resembling the less fibrous architectures seen in Samples 1–2, and another consisting of finer, more densely packed bundles characteristic of the more highly aligned structures in Samples 4–5. This within‐sample heterogeneity represents natural variation in the intended structure rather than measurement noise and is therefore reflected in the observed SD values, which were also previously shown by Ma et al. (2024). Overall, the results confirm that the sample set spans a broad and intentionally variable range of macrostructural fibrousness, providing a suitable foundation for subsequent analyses linking instrumental structure to sensory perception (Figure 2).

### 3.2 Mechanical Properties and Serum Release in Compression

**FIGURE 3:**
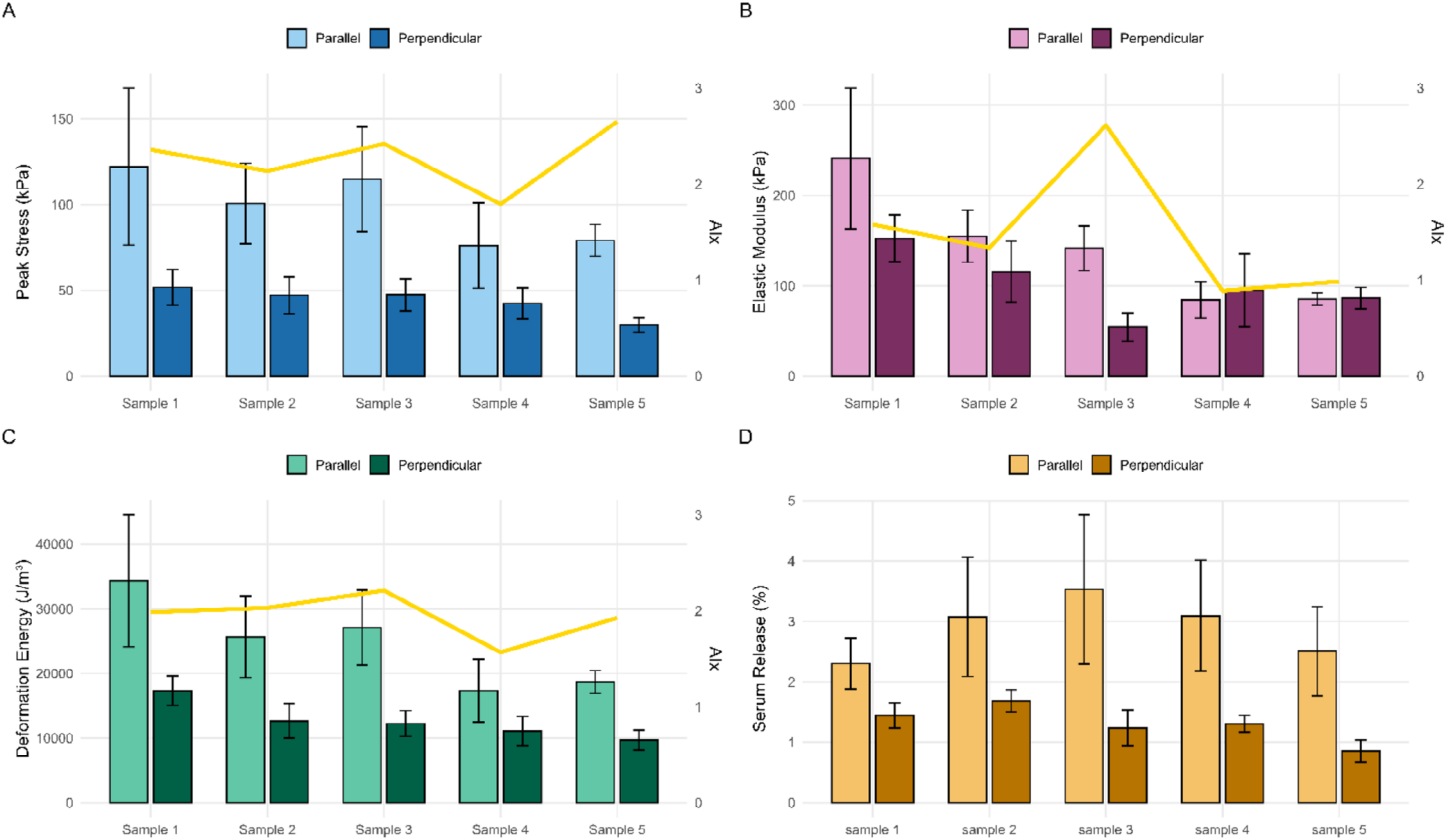
(A) Peak stress, (B) Elastic Modulus, (C) Deformation Energy, and (D) Serum Release during compression testing for the five samples measured parallel (light color) and perpendicular (dark color) to the fiber direction. Secondary axis provides the anisotropy index of the mechanical parameters (yellow line).

Directional compression testing using a texture analyzer was performed to study the mechanical properties and serum release of the samples. Representative stress–strain curves (Appendix S4) revealed differences in mechanical behavior between orientations. Parallel specimens showed strain stiffening, which is consistent with progressive fiber engagement, while perpendicular specimens showed strain weakening, reflecting reduced structural contribution of fibers across this axis. Additionally, parallel specimens of samples 1 and 2 seem to break at a lower strain than samples 3, 4, and 5. Peak stress, Elastic Modulus, and Deformation Energy progressively decreased from samples 1 to 5 (Figure 3A–C). For all three parameters, values measured parallel to the fiber direction were generally higher than those measured perpendicular to the fiber direction. This directional dependence was more pronounced for peak stress and deformation energy than for elastic modulus.

The calculated anisotropy index (AIx) exceeded 1 for all samples and parameters, confirming mechanical anisotropy. However, the magnitude of anisotropy depended on the mechanical parameter considered. Peak stress exhibited pronounced anisotropy across all samples, including samples 4 and 5 (Figure 3A). In contrast, elastic modulus anisotropy decreased along the sample series, with AIx values approaching 1 for samples 4 and 5, indicating near‐isotropic stiffness (Figure 3B). Sample 3 showed the highest AIx for elastic modulus.

Deformation energy also decreased from sample 1 to sample 5 and remained direction‐dependent across all samples (Figure 3C), although anisotropy was reduced in the later samples.

Serum release, measured using a filter paper absorption method, varied between samples, indicating differences in fluid expulsion behavior under mechanical deformation (Figure 3D). Sample 3 exhibited the highest serum release under compression parallel to the fiber direction, whereas sample 1 exhibited the lowest. Across all samples, compression parallel to the fiber direction consistently resulted in higher serum release than perpendicular compression.

### 3.3 Descriptive Analysis

The evaluation revealed significant differences (*p* < 0.001) between two or more samples in all attributes across all chewing phases (Table 2). Further, no replicate or higher‐order interaction effects were significant (*p* > 0.05), indicating stable panel performance. Only minor and transient assessor effects were detected specifically for attributes *Juicy* (5 chews) and *Fibrous* (10 chews), and a sample × assessor interaction for *Chewiness* (Appendix S5).

**TABLE 2:** Mean ± SD intensity scores (15 cm line scale) for DA attributes of the five samples evaluated by the trained panel.

Attributes (chews)	Sample 1	Sample 2	Sample 3	Sample 4	Sample 5
	Mean (SD)	Mean (SD)	Mean (SD)	Mean (SD)	Mean (SD)
Visual					
Visible Fibrous (0)	0.9^a^ (1.7)	2.1^a^ (3.0)	8.4^b^ (3.7)	11.2^c^ (3.6)	14.6^d^ (0.8)
Oral—First bite					
Hardness (1)	14.3^a^ (1.1)	11.9^b^ (2.8)	6.8^c^ (4.1)	3.9^d^ (4.1)	1.6^e^ (3.1)
Springiness (1)	14.3^a^ (1.3)	12.1^b^ (2.5)	6.5^c^ (4.0)	4.1^d^ (4.1)	1.6^e^ (3.2)
Denseness (1)	14.4^a^ (1.0)	12.0^b^ (3.0)	6.9^c^ (3.9)	4.1^d^ (4.0)	1.5^e^ (3.2)
Cohesiveness (2)	1.0^a^ (1.6)	2.7^a^ (2.9)	8.0^b^ (3.9)	10.21^c^ (4.5)	12.9^d^ (4.2)
Oral—Chewing phase					
Fibrous (5)	0.9^a^ (1.6)	2.6^a^ (2.6)	8.1^b^ (3.8)	10.7^c^ (4.2)	13.5^d^ (3.1)
Juicy (5)	1.9^a^ (2.8)	3.8^a^ (3.6)	9.2^b^ (3.6)	11.1^bc^ (4.0)	12.9^c^ (3.0)
Oral—End phase					
Chewiness (10)	2.4^a^ (4.3)	4.0^a^ (4.3)	7.4^b^ (3.9)	9.3^b^ (4.9)	12.1^c^ (4.9)
Fibrous (10)	1.0^a^ (1.8)	2.6^a^ (2.5)	8.5^b^ (3.7)	10.9^c^ (4.0)	13.4^d^ (3.1)

*Note:* Within rows, means with the same letter do not differ significantly (Tukey, *p* < 0.001). Attribute labels with numbers (1, 2, 5, 10) denote the number of chews at which evaluation was made. *Visible fibrousness* was assessed prior to tasting and only in the visual condition.

*Visible fibrousness* differed significantly among samples (*p* < 0.001). Samples 1 (0.9) and Sample 2 (1.9) scored significantly lower than Sample 3 (8.4), which was lower than Sample 4 (11.2), and Sample 5 (14.6) showed the highest score for *visible fibrousness*. *Oral fibrousness* during mastication followed the same trend at both chew 5 and chew 10, with chew 5 scores ranging from 0.8 in Sample 1 to 13.4 in Sample 5, and chew 10 scores from 1.0 to 13.4. Although *oral fibrousness* increased slightly between chew 5 and chew 10, though not significant, indicating that perceived fibrousness did not change appreciably between these chewing stages for the samples studied.

The texture attributes showed two opposing trends with increasing *fibrousness*. *Hardness, springiness*, and *denseness* decreased markedly from Sample 1 to Sample 5, with clear and significant separation between all samples. *Hardness* declined from Sample 1 (14.3) to Sample 5 (1.6), and *springiness* followed the same pattern, decreasing from Sample 1 (14.3) to Sample 5 (1.6). *Denseness* likewise decreased from Sample 1 (14.4) to Sample 5 (1.5), with all pairwise differences significant (*p* < 0.001).

In contrast, *cohesiveness, fibrousness, juiciness*, and *chewiness* increased across samples but exhibited less uniform discrimination. *Cohesiveness* increased from Sample 1 (1.0) and Sample 2 (2.8), which did not differ significantly, to intermediate levels in Sample 3 (7.9) and the highest values in Sample 4 (10.2) and Sample 5 (12.9). *Oral fibrousness* increased progressively across samples, with Sample 1 and Sample 2 again not differing significantly, while Samples 4 and 5 formed the highest group.

*Juiciness* increased from Sample 1 (2.8) to Sample 5 (13.0), although no significant difference was observed between Sample 4 and Sample 5. *Chewiness* likewise increased from Sample 1 (2.3) to Sample 5 (12.0); however, Sample 3 and Sample 4 did not differ significantly, whereas Sample 5 was clearly distinct. Overall, increasing from Sample 1 to Sample 5 shifted the sensory profile from hard, springy, and dense textures toward products perceived as more cohesive, fibrous, juicy, and chewy, while also revealing differences in the extent to which attributes discriminated among adjacent samples.

### 3.4 Effect of Evaluation Condition (Visual vs. Blindfolded)

A key aspect of the experimental design was the comparison between visual and blindfolded evaluation conditions to determine the influence of visual input on texture perception. During the visual condition, assessors assessed both visible fibrousness and the oral texture attributes. In contrast, the blindfolded condition evaluated only the oral texture attributes, with visual cues completely eliminated.

**FIGURE 4:**
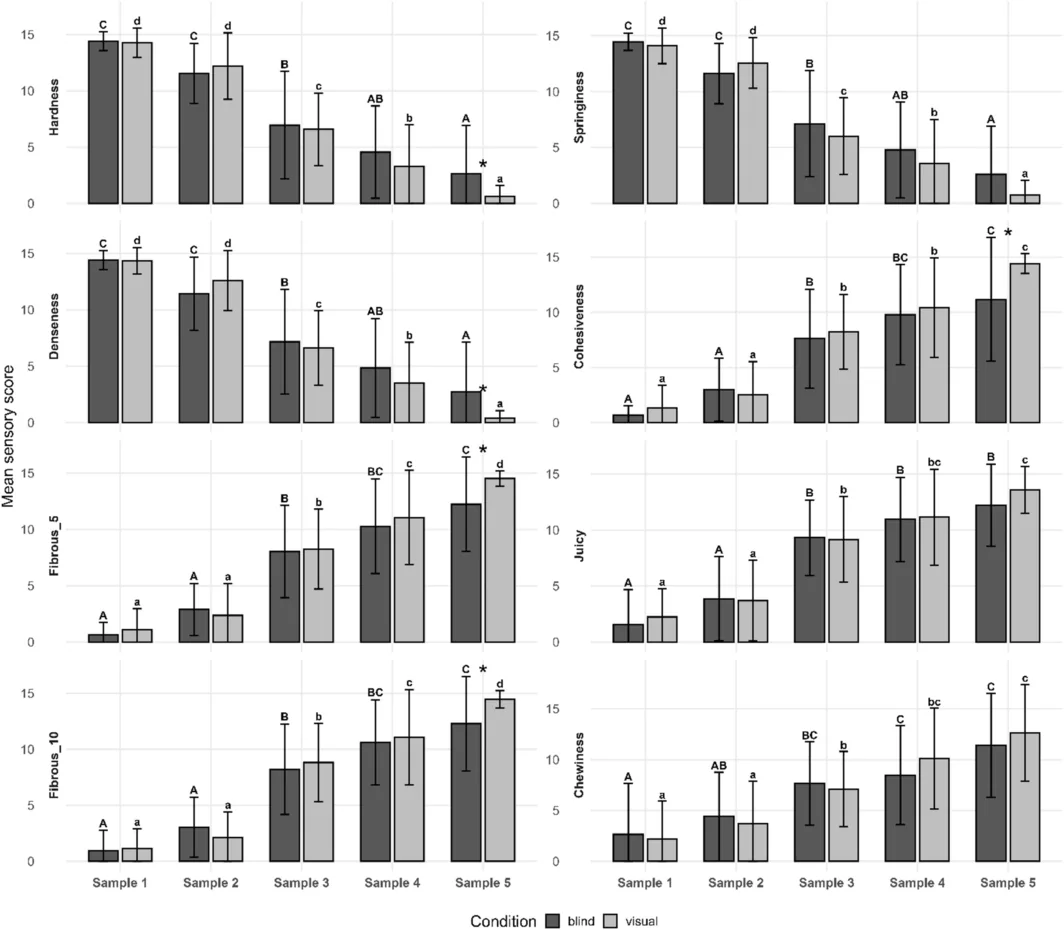
Mean sensory descriptive analysis (DA) ratings (±SD) for texture attributes evaluated under visual and blindfolded conditions across samples. Bars represent mean scores and error bars indicate standard deviations. Uppercase letters (A–C) denote significant differences between samples within the blindfolded condition, while lowercase letters (a–d) denote significant differences between samples within the visual condition (Tukey's HSD, *p* < 0.001). Asterisks (*) indicate significant differences between visual and blindfolded evaluation conditions for the same sample and attribute (*p* < 0.05).

Direct comparison of visual and blind evaluations showed that significant differences occurred only for Sample 5, the most fibrous formulation, and only for a subset of attributes: *hardness, springiness, denseness, cohesiveness*, and *fibrousness*, at both chew 5 and chew 10 (Figure 4). For all other samples and attributes, ratings did not differ significantly between conditions. These findings indicate that the influence of visual input on sensory ratings was confined to the highest fiber sample and became apparent primarily at higher intensity levels of texture perception.

Under blindfolded conditions, assessors used a narrower portion of the upper end of the 15 cm line scale compared with the visual condition. As a result, ratings for the most fibrous samples (Samples 4 and 5) remained lower under blindfolded evaluation than under visual evaluation, leading to compressed scoring patterns at higher intensity levels (Figure 4). Consequently, the relative distance between Samples 4 and 5 was reduced, and no significant differences were detected between these two samples for any attribute under blindfolded conditions.

When visual cues were available, ratings extended further toward the upper end of the scale, and Samples 4 and 5 were clearly discriminated for most attributes. *Juiciness* and *chewiness* were exceptions, for which no significant differences were observed between these two samples under either condition. Greater variability in ratings was observed under blindfolded conditions, as reflected by consistently higher standard deviations across most attributes, particularly for Sample 5 (Figure 4). These results indicate reduced agreement among assessors and greater uncertainty in texture evaluation when visual information was unavailable, especially for the most fibrous formulation.

Fibrous structure was present in all samples, consistent with the intended biomimetic variability of the structures, but was most pronounced in Sample 3. As shown in Figure 2, Sample 3 contained two distinct fiber layers within a single piece, with one section composed of thicker, plate‐like fibers similar to the less fibrous structures observed in Samples 1 and 2 and another region containing finer, more densely packed fiber bundles similar to the highly fibrous structures observed in Samples 4 and 5, rather than a uniform fiber structure throughout the sample.

### 3.5 Integration of Sensory and Instrumental Data

**FIGURE 5:**
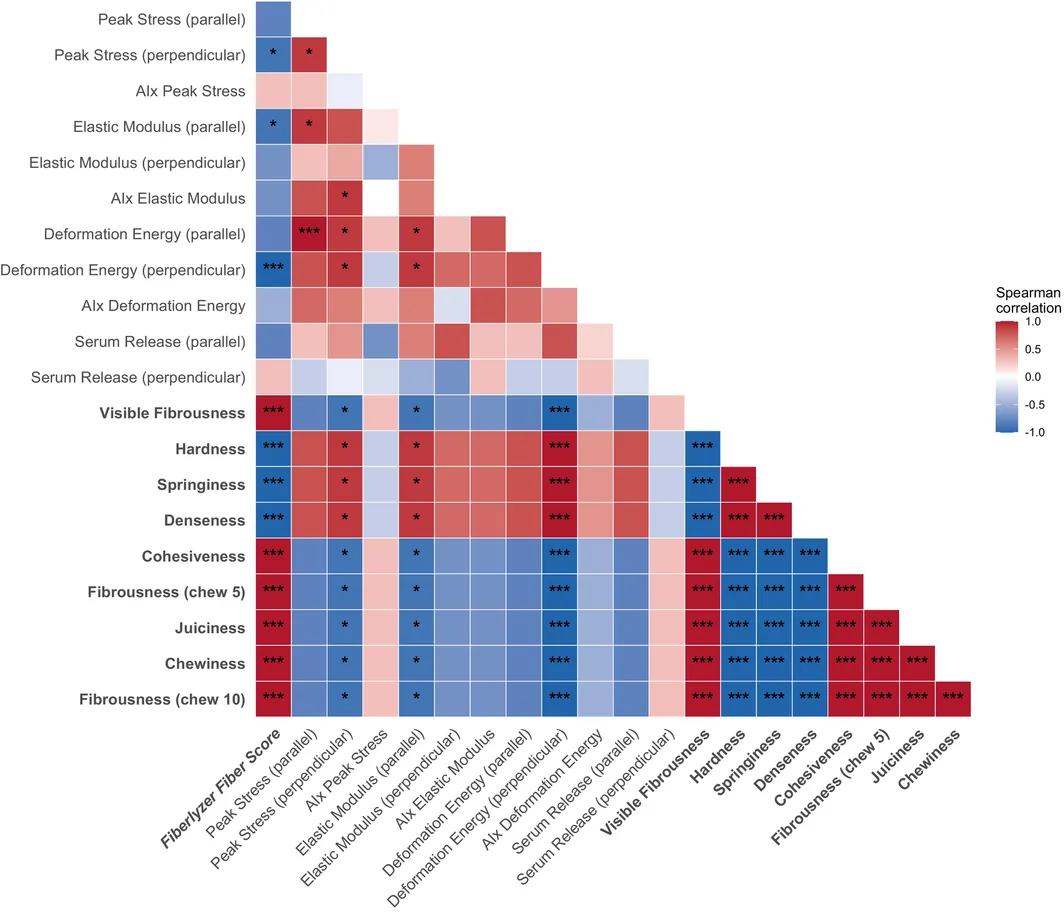
Spearman correlation heatmap showing relationships between Fiber Score, instrumental structural measurements, moisture loss, and sensory attributes. Color intensity represents the strength and direction of correlations (Spearman's correlation coefficient, ρ; blue = negative, red = positive). Only the lower triangle is displayed. Sensory attributes are shown in bold text to distinguish them from instrumental variables. **Asterisks indicate statistically significant correlations (*p < 0.05, **p < 0.01, *p < 0.001); cells without asterisks were not statistically significant.

To provide an overview of relationships among variables, a Spearman correlation heatmap was generated (Figure 5), showing associations between Fiber Score, instrumental measurements, and sensory attributes. Strong positive correlations were observed among fibrousness‐related sensory attributes across chewing stages, indicating internal consistency of fibrousness perception.

Fiber Score showed positive correlations with several instrumental mechanical parameters, particularly those measured in the parallel direction, as well as with sensory fibrousness. Instrumental mechanical parameters, Peak Stress, Elastic Modulus, and Deformation Energy, were strongly correlated with each other, and measurements obtained in the parallel and perpendicular directions clustered together with their corresponding anisotropy indices.

Sensory *hardness* and *chewiness* were positively correlated with multiple instrumental mechanical parameters, including Peak Stress, Elastic Modulus, and Deformation Energy, indicating that these attributes co‐varied with resistance to deformation and fracture. Sensory *fibrousness*, assessed at both chew 5 and chew 10, also showed positive correlations with several mechanical parameters and with the Fiber Score, reflecting consistency between instrumental structure metrics and perceived fibrous texture across chewing stages.

In contrast, sensory *juiciness* and *cohesiveness* exhibited weaker and more selective correlations with instrumental measures and lower correlations with the Fiber Score, suggesting that these attributes were less directly associated with the measured mechanical properties. Serum release showed weak to moderate correlations with both instrumental and sensory attributes, indicating that moisture‐related perceptions followed patterns distinct from *hardness, chewiness, springiness, denseness*, and *fibrousness* related sensory attributes.

To further examine the relationship between structural fibrousness and sensory perception, analyses focused on the association between the computed Fiber Score and sensory fibrousness attributes. Correlation analysis was used to quantify the strength of association between Fiber Score and sensory perceived fibrousness, while regression analysis was applied to evaluate whether Fiber Score could predict perceived fibrousness during oral processing. Sensory and instrumental datasets were therefore analyzed jointly to determine whether the Fiber Score, which quantifies visible fibrousness, was systematically related to and predictive of oral fibrousness perception.

**FIGURE 6:**
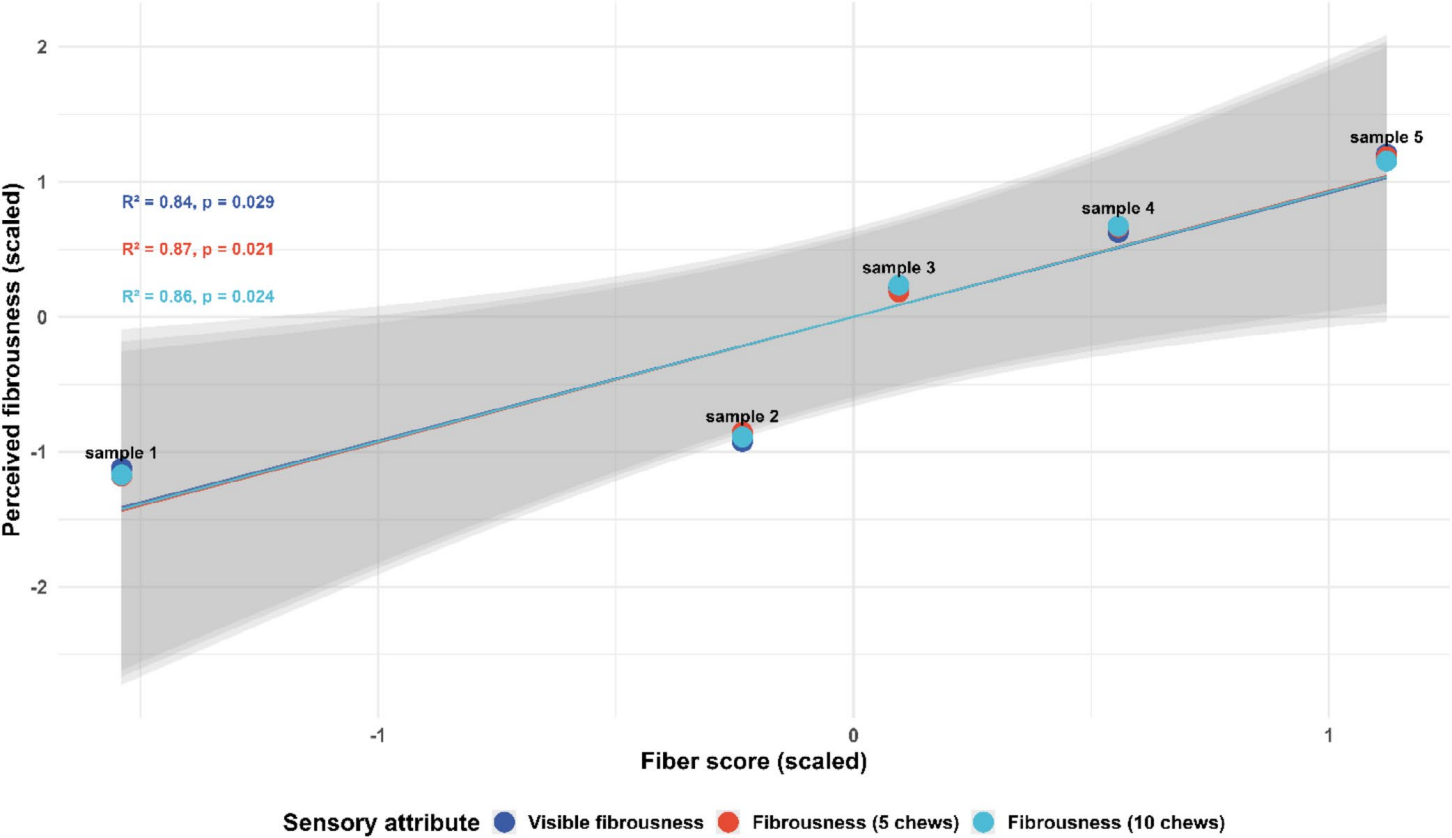
Relationship between instrumental Fiber Score and perceived fibrousness attributes. *Visible fibrousness* was evaluated during the descriptive analysis visual condition, while *fibrousness* after 5 and 10 chews represent combined scores from visual and blindfolded evaluations. Solid lines represent least‐squares regression fits. Gray shaded areas indicate 95% confidence intervals. *R* ^2^ and *p*‐values for each regression are shown in the plot.

The correlation heatmap revealed strong positive associations between instrumental Fiber Score and all sensory fibrousness attributes. Based on these correlations, linear regression analyses were performed to quantify the relationships between the Fiber Score and sensory perceived *fibrousness* (Figure 6).

The Fiber Score was a strong predictor of *visible fibrousness* (*R* ^2^ = 0.84, *p* = 0.029), *fibrousness* after 5 chews (*R* ^2^ = 0.87, *p* = 0.021), and *fibrousness* after 10 chews (*R* ^2^ = 0.86, *p* = 0.024). These relationships were consistent across both visual and blindfolded evaluation conditions, indicating that oral perception of fibrousness was largely independent of visual access to the sample (see Figure 4).

When analyzed for visual and blindfolded conditions separately, the Fiber Score remained a strong predictor of perceived fibrousness under both visual and blindfolded conditions. Under the visual condition, correlations were observed for fibrousness after 5 chews (*R* ^2^ = 0.85, *p* = 0.026) and after 10 chews (*R* ^2^ = 0.83, *p* = 0.031). Under the blindfolded condition, slightly stronger relationships were found for fibrousness after 5 chews (*R* ^2^ = 0.89, *p* = 0.016) and after 10 chews (*R* ^2^ = 0.88, *p* = 0.018). Sample 2 consistently showed the largest deviation from the fitted regression lines across all fibrousness attributes, suggesting a structural–sensory mismatch for this formulation.

To examine the multivariate relationship between fibrous structural predictors derived from image‐based Fiber Score analysis, instrumental mechanical predictors obtained from directional compression testing, and sensory perception, a partial least squares (PLS) regression model was applied. The first two PLS components accounted for 72.6% and 27.4% of the explained variance in the predictor matrix (X), respectively. In the sensory response matrix (Y), the first two components explained 91.6% and 4.1% of the variance (*R* ^2^‐based, averaged across sensory attributes), respectively, indicating that the predictive relationship between structural predictors and sensory perception is overwhelmingly dominated by the first dimension. As a result, nearly all explainable sensory information is captured by the first PLS component alone, with the second component contributing only marginal additional predictive value.

**FIGURE 7:**
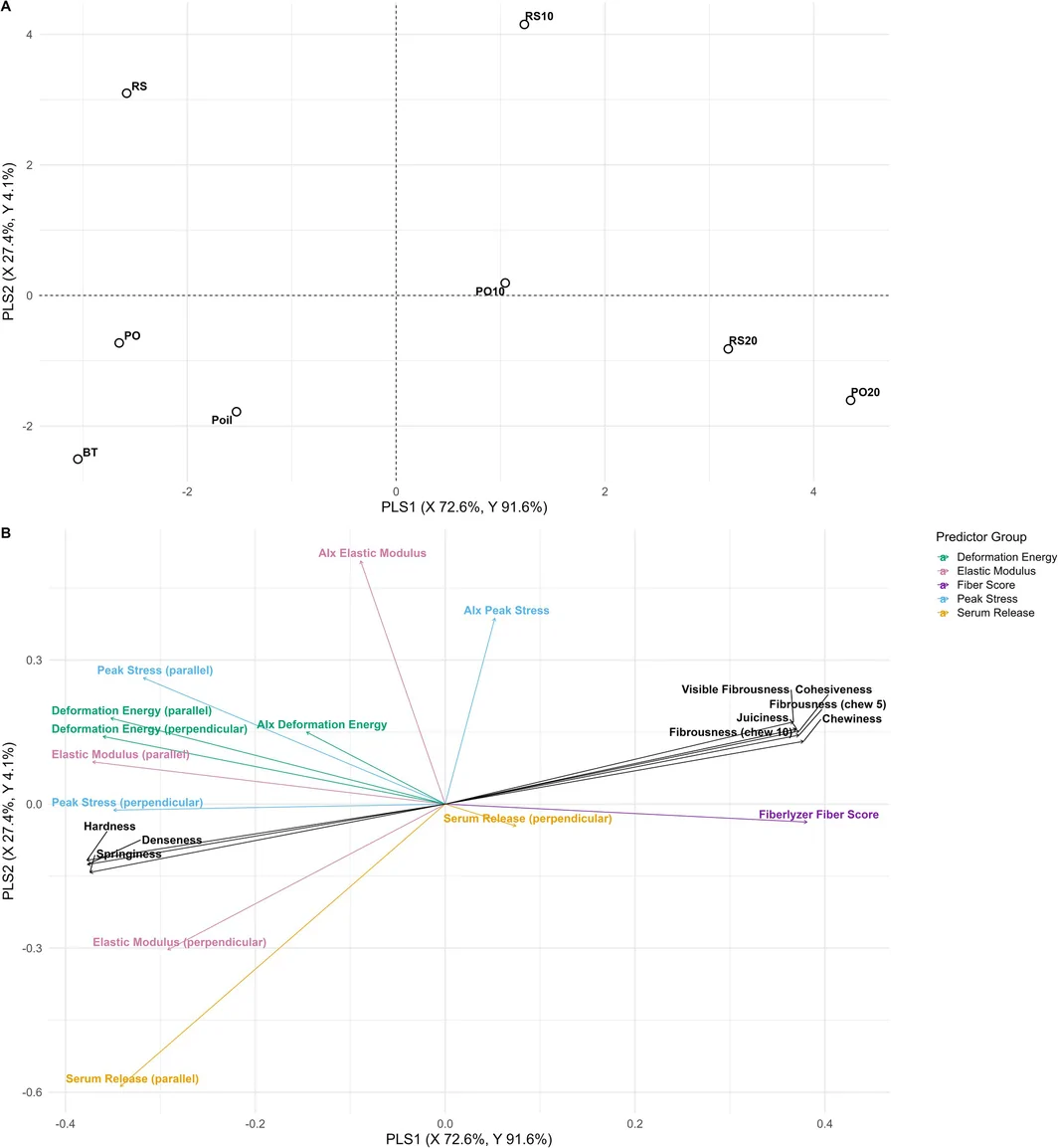
PLSR scores and loadings plots illustrate the multivariate relationships between instrumental predictors (X‐block: Fiber Score, Deformation Energy, Elastic Modulus, Peak Stress, and Moisture Loss) and sensory response variables (Y‐block: Sensory texture attributes). A: The score plot with samples. B: Predictor and response loadings are projected onto the first two PLS components.

The PLSR plot (Figure 7) further shows that sensory *fibrousness, cohesiveness, chewiness, hardness*, and *juiciness* cluster together and project strongly along the positive direction of PLS1, suggesting that the structural modifications introduced across the sample set influenced several related sensory texture attributes simultaneously, resulting in substantial covariance among the sensory variables. Several instrumental predictors aligned with this same direction, including Peak Stress (perpendicular), Deformation Energy (perpendicular and parallel), Elastic Modulus (perpendicular), and the anisotropy indices for Deformation Energy. These instrumental parameters were strongly associated with the sensory attributes located in this region of the PLSR space, indicating close relationships between structural, mechanical, and sensory properties.

The Fiber Score also projected positively along the first PLS component confirming its strong association with perceived *fibrousness* and related sensory attributes. By contrast, Serum Release variables (Serum release (perpendicular and parallel)) projected in the opposite direction of the first PLS component. PLS2 captured secondary variation mainly associated with directional differences in instrumental mechanical parameters and serum release behavior, but contributed little to differentiation of sensory attributes.

**FIGURE 8:**
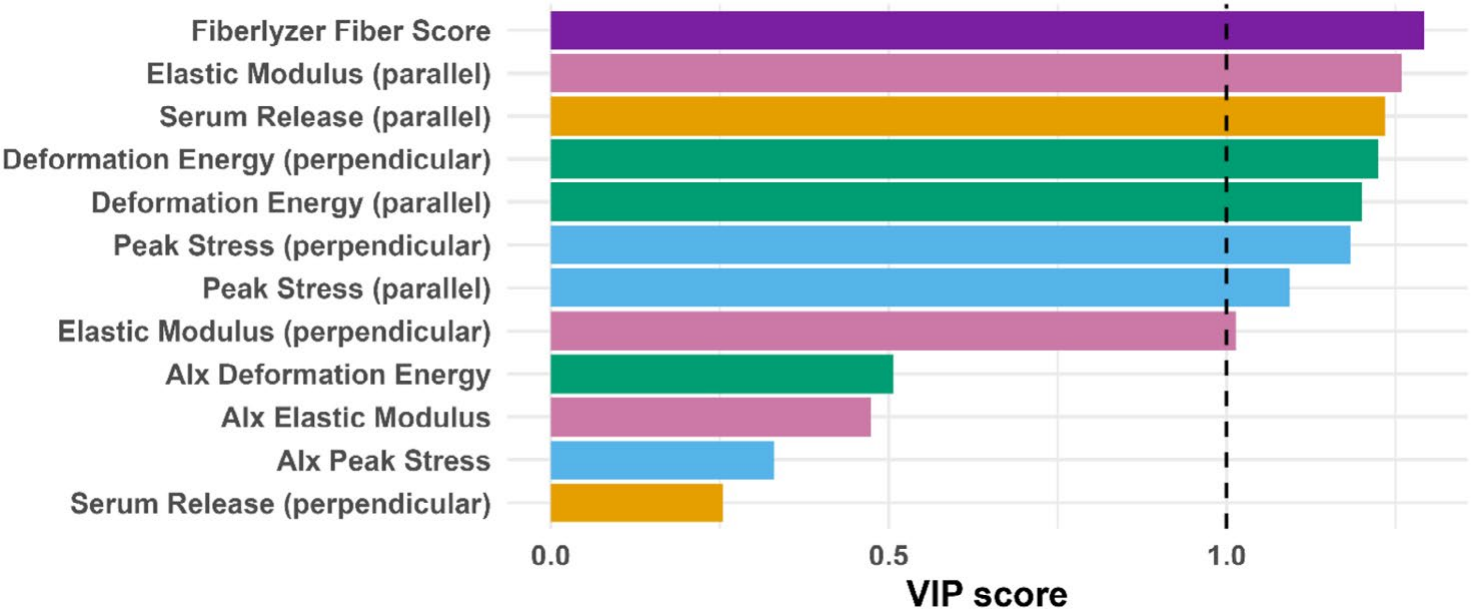
Variable Importance in Projection (VIP) scores from the Partial Least Squares Regression (PLSR) model. VIP values indicate the relative contribution of instrumental predictors to explaining variation in sensory texture attributes. Variables with VIP ≥ 1 are considered important contributors to the structure–sensory relationship.

To quantitatively assess the contribution of individual predictors beyond qualitative inspection of the biplot, Variable Importance in Projection (VIP) scores were calculated. The highest VIP values (VIP > 1) were observed for the Fiber Score, Serum Release (perpendicular), Elastic Modulus (perpendicular), Deformation Energy (both directions), and Peak Stress (both directions), indicating that fiber‐related properties, serum release, and key mechanical attributes were all dominant contributors to the structure–sensory relationships captured by the model (Figure 8).

## 4 Discussion

### 4.1 Macrostructural Fibrous Structure in Relations to Sensory Perception of Fibrousness

To address the first study aim, the relationship between controlled variation in fibrous structure and sensory perception of fibrousness was examined. Systematic variation in fibrous structure successfully modulated perceived fibrousness in plant‐based meat analogues, demonstrating that macrostructural fiber organization is a key determinant of oral fibrousness perception. The Fiberlyzer Fiber Score showed strong correlations with sensory fibrousness assessed during visual inspection (Visible Fibrous) and during oral processing (Fibrous after both 5 and 10 chews) (Figure 6), indicating that image‐derived macrostructural fibrousness provides a robust proxy under the tested conditions for perceived fibrousness. The similar relationships observed at both chewing stages further suggest that structures responsible for fibrous perception remain perceptually salient throughout the evaluated stages of mastication despite progressive structural breakdown.

These findings support the established concept that aligned protein structures give rise to anisotropic, muscle‐like fracture and tearing behavior, which underpins fibrous texture perception (Dekkers et al. 2018; Schreuders et al. 2021; Zink et al. 2024). Complementary structural studies have further demonstrated that fiber alignment and mechanical anisotropy can be systematically controlled through formulation and processing conditions in extrusion‐ and shear‐based systems (Beniwal et al. 2021; Hwang et al. 2024; Taghian Dinani et al. 2023). However, most of this prior work has focused on structural or mechanical characterization, without directly testing whether such controlled variation in fibrous structure translates into predictable differences in sensory fibrousness perception. The present results provide direct sensory evidence that macrostructural fiber alignment is not only a processing outcome, but a sensory‐relevant design parameter that governs how fibrousness is perceived during eating.

Minor deviations between computed Fiber Score and sensory fibrousness, such as those observed for Sample 2, are expected and can be explained by differences between instrumental image acquisition and sensory evaluation conditions. In other words, Fiberlyzer quantifies fibrousness from samples that are bent open to expose internal structure. In contrast, *sensory fibrousness* is judged visually on unmanipulated samples, reflecting only the structural features accessible to direct observation by the panel. In general, the influence of sample presentation and structural exposure on visual texture assessment is well recognized in sensory studies (Nishinari and Fang 2018; van Vliet 1996).

Accordingly, correspondence between instrumental fiber alignment metrics and visual sensory ratings depends on the extent to which fibrous structures are externally discernible. From an applications perspective, therefore, macrostructural image‐based methods such as Fiberlyzer are particularly well suited for the development and evaluation of fibrous PBMA concepts that aim to replicate whole‐cut muscle structures, including beef‐, pork‐, poultry‐, or fish‐like products, where fibrous architecture is a defining sensory feature.

In contrast, in PBMA formats lacking visible or exposable fiber strands, such as burger patties, macrostructural fiber metrics are less likely to provide informative discrimination, even when small sensory differences are perceived. This highlights that while instrumental and sensory approaches are complementary, trained sensory evaluation remains essential for interpreting structure–perception relationships, reflecting the distinction between material texture quantified instrumentally and sensory texture emerging during oral processing (Chen 2026; Nishinari and Fang 2018). Within this framework, Fiberlyzer is best positioned as a complementary reference tool supporting sensory interpretation when fibrousness is a key product attribute.

### 4.2 Effect of Visual Cues on Fibrousness and Texture Perception: Visual Versus Blindfolded Evaluations

To address the second study aim, sensory ratings obtained under visual and blindfolded conditions were compared to assess how visual cues influence fibrousness perception and texture evaluation. Although visual–oral interactions are widely acknowledged in sensory science, their specific role in the perception of fibrousness in PBMAs has rarely been isolated experimentally. Texture perception in fibrous plant‐based meat analogues is inherently multimodal, integrating visual, tactile, and oral cues, as explicitly considered in trained sensory studies that distinguish visual texture, manual texture, and mouthfeel attributes (e.g., Barnés‐Calle et al. 2024). In contrast to these studies, the present design explicitly decoupled visual input from oral processing, allowing the contribution of visual cues to be assessed independently of oral fibrousness perception.

Comparison of visual and blindfolded evaluation conditions showed that panelists were able to perceive and discriminate texture differences without visual input, demonstrating that oral tactile cues alone are sufficient for fibrousness perception once assessors are trained on the attribute definition. However, removal of visual cues altered scale use and agreement, particularly at higher intensity levels. This behavior aligns with sensory theory describing multimodal integration and increased response variability when visual calibration cues are absent (Lawless and Heymann 2010). In the present study, assessors were trained under visual evaluation conditions, whereas blindfolded evaluation was introduced only during the final session. This training asymmetry likely amplified reliance on visual reference points for scale anchoring, contributing to increased uncertainty once visual input was removed. Importantly, this effect was not uniform across samples: evaluation condition significantly affected ratings only for the most fibrous formulation (Sample 5) and primarily for high‐intensity attributes, while lower‐intensity samples showed no systematic differences between visual and blindfolded conditions. This indicates that visual cues primarily modulate intensity calibration rather than the basic detectability of fibrousness.

Local variation in fibrous structure within individual samples further helps explain differences in response variability across evaluation conditions. For instance, we observed during handling and image inspection that several samples exhibited visually apparent regions of more and less pronounced fibrous alignment. Such spatial variation in fibrous organization is a common feature of processed soft solids and fibrous protein networks, where local differences influence deformation and fracture behavior (van Vliet 1996). Under visual evaluation, assessors could recognize and contextualize these local alignment differences, supporting more consistent scale use. Under blindfolded conditions, reliance on oral tactile cues alone increased inter‐individual variability when such structural variation was encountered unpredictably during mastication. This is also a likely explanation for the observed differences between sensory and instrumental measurement modes: image‐based or mechanical methods interrogate specific regions, whereas sensory perception integrates deformation and fracture across multiple contacts and chewing cycles (Nishinari et al. 2019; Nishinari and Fang 2018). For samples with more pronounced internal layering of fibrous structure, such as Sample 3 (Figure 2), relatively higher variability in sensory ratings was observed, particularly under blindfolded evaluation. This pattern suggests that spatial variation in fibrous organization may contribute to increased response variability when assessors rely solely on oral tactile cues, highlighting a potential interaction between structural organization and sensory modality.

Together, these results demonstrate that while fibrous perception does not depend on visual input per se, visual access to fibrous structure plays a critical role in scale calibration, response consistency, and discrimination at higher intensity levels. This study therefore provides direct experimental evidence that visual cues primarily influence how fibrousness is judged, rather than whether it is perceived, extending existing multimodal texture theory to fibrous PBMAs.

### 4.3 Instrumental Measures of Fibrous Structure and Mechanical Anisotropy in Relation to Sensory Perception

To address the third study aim, relationships between macrostructural fibrous structure quantified by the Fiber Score, instrumental mechanical anisotropy assessed by directional compression testing, and perceived sensory texture attributes were examined using correlation analysis and PLSR modeling. Multivariate analysis demonstrated that macrostructural fibrous structure, quantified by the image‐derived Fiber Score, is the strongest instrumental contributor among the tested predictors of perceived fibrousness. VIP analysis from the PLSR model showed that the Fiber Score contributed more strongly to sensory texture prediction than any individual instrumental mechanical compression parameter. This provides quantitative evidence within the present experimental framework that visible macrostructural fibrous structure is more critical for fibrousness perception than bulk mechanical resistance alone.

Directional compression testing confirmed that samples exhibiting higher sensory fibrousness also showed pronounced mechanical anisotropy, particularly in parameters describing post‐yield deformation and fracture such as Peak Stress and Deformation Energy. Across correlation and multivariate analyses, these large‐deformation metrics aligned more consistently with sensory fibrousness than small‐strain elastic parameters, indicating that perceived fibrousness is governed primarily by fracture behavior and fiber separation during oral breakdown rather than by stiffness of the intact structure. Such anisotropic fracture responses are characteristic of aligned fiber networks in which load transfer and interfacial bonding dictate crack propagation pathways (Bourne 2002; van Vliet 1996) and have been widely reported in extrusion‐ and shear‐structured plant‐protein systems (Beniwal et al. 2021; Hwang et al. 2024; Taghian Dinani et al. 2023). These results therefore support oral‐processing frameworks emphasizing that texture perception in solid foods emerges mainly from large‐deformation comminution events during (Chen 2009; Nishinari and Fang 2018; Szczesniak 2002).

At the same time, the results demonstrate that fiber alignment alone is insufficient to guarantee consistent sensory perception. Differences between samples indicated that macrostructural continuity and inter‐fiber bonding modulate how anisotropic structures fracture during compression and chewing. For example, the layered organization observed in Sample 3 produced pronounced directional differences in elastic response and increased variability in both instrumental and sensory measurements, whereas the low deformation energy observed for Sample 5 despite its fibrous appearance suggested weak inter‐fiber connectivity and rapid separation along fiber interfaces. Comparable observations regarding the importance of fiber packing density and network integrity have been reported in structured plant‐protein matrices (Beniwal et al. 2021; Hwang et al. 2024; Taghian Dinani et al. 2023). These findings indicate that instrumental prediction of fibrousness depends not only on visible alignment but also on the functional continuity of the fibrous network governing fracture pathways during oral processing.

In contrast to structural and fracture‐related predictors, instrumental serum release showed weaker and more selective associations with sensory attributes, indicating that lubrication‐related perceptions operate within a partially independent perceptual domain. This observation is consistent with oral‐processing concepts describing juiciness perception as emerging from progressive saliva incorporation and dynamic lubrication during mastication rather than from fluid expulsion under externally imposed compression (Chen 2009; Nishinari and Fang 2018). Supporting this interpretation, previous studies have reported limited or even inverse relationships between compression‐induced fluid release and perceived juiciness (Rudge et al. 2025; Xue et al. 2025). Within the present experimental framework, serum release therefore provided contextual information about moisture‐related mouthfeel but did not function as a direct predictor of fibrousness perception.

Taken together, the results suggest a hierarchical relationship between instrumental measures and sensory perception in fibrous plant‐based matrices. Macrostructural alignment primarily determines whether a product is perceived as fibrous, while large‐deformation mechanical anisotropy explains how that fibrous structure breaks down during chewing. Moisture‐related instrumental metrics, by contrast, contribute to secondary mouthfeel dimensions associated with lubrication and cohesiveness. This differentiation highlights the importance of selecting instrumental approaches according to the specific sensory construct of interest.

From an application perspective, these findings indicate that macrostructural fiber alignment should be treated as a primary design parameter in PBMA formulation, while directional mechanical testing is most informative for understanding fracture behavior and texture breakdown during eating rather than for predicting fibrousness per se. Image‐based methods such as Fiberlyzer therefore show strong potential as rapid screening tools during product development, particularly once structure–perception relationships have been established.

Importantly, this study provides one of the first quantitative demonstrations, based on trained descriptive sensory analysis, that an image‐based macrostructural descriptor can outperform conventional mechanical texture metrics in predicting sensory fibrousness. By integrating Fiberlyzer analysis with directional compression testing and trained sensory evaluation, the present work establishes a clear structure–mechanics–perception framework for fibrous PBMAs, enabling instrumental measures to be interpreted in a sensory‐relevant context rather than in isolation.

### 4.4 Limitation and Future Studies

In this study, directional single cycle compression testing was used as a targeted instrumental mechanical probe to characterize fiber controlled fracture and load bearing behavior and to relate these properties to sensory perception of fibrousness. While this approach enabled clear and interpretable links between macrostructural fiber structure (Fiber Score), mechanical anisotropy, and sensory texture attributes, it captures only a subset of the deformation modes involved during mastication. Specifically, uniaxial compression primarily probes resistance to bulk deformation and fracture initiation, but does not capture tensile, shear, or combined deformation modes that are particularly relevant for strand separation, fiber pull‐apart, and tearing behavior in fibrous structures. Future studies could extend this by incorporating complementary deformation modes, such as tensile testing, shear‐dominated tests, or combined compression–shear geometries, which may better represent the mechanical interactions governing fiber separation during chewing.

In addition, instrumental measurements in the present study were limited to single compression cycles, whereas oral processing involves repeated, cyclic deformation accompanied by progressive structural breakdown and fluid redistribution. Although repeated breakdown was addressed sensorially by evaluating fibrousness and juiciness after defined chewing stages (5 and 10 chews), corresponding cyclic or multi‐stage instrumental testing was not performed. Furthermore, the structural modifications introduced across the sample set influenced multiple sensory and mechanical attributes simultaneously, resulting in covariance among several of the measured variables. Future work could combine repeated or cyclic mechanical loading with time‐resolved sensory evaluation to provide deeper insight into how fibrous structures evolve during mastication and how fluid mobility develops across chewing cycles.

Finally, integrating dynamic oral‐processing simulations that include saliva addition, temperature control, and multi‐axial deformation would further improve the physiological relevance of instrumental measurements. Such approaches would strengthen structure mechanics perception models and support the rational design of next‐generation plant‐based meat analogues with targeted fibrous texture and juiciness profiles.

## 5 Conclusion

This study investigated how macrostructural fibrous structure, instrumental mechanical behavior, and sensory perception are related in plant‐based meat analogues by combining image‐based Fiber Score analysis, directional compression testing, and trained descriptive sensory evaluation.

The most important conclusions are as follows: first, controlled variation in fibrous structure resulted in systematic differences in perceived texture. Higher image‐derived Fiber Scores were consistently associated with higher sensory ratings of fibrousness during mastication, demonstrating that macrostructural fiber alignment quantified from macroscopic images closely relates to perceived fibrous texture.

Second, removing visual cues showed that fibrousness can be reliably perceived through oral processing alone. However, differences between visual and blindfolded evaluations for the most fibrous samples indicate that visual structure cues can influence the perceived intensity of fibrousness when structural alignment is high, modulating but not replacing the underlying structure‐driven oral perception.

Third, instrumental measurements differed in their ability to explain sensory perception. Image‐derived Fiber Score showed the strongest relationship with sensory fibrousness, whereas directional compression measurements provided complementary information on fracture resistance and structural integrity related to hardness, chewiness, and cohesiveness. In contrast, serum release during compression showed relatively weak associations with the sensory attributes, suggesting that instrumental fluid release alone cannot fully explain perceived juiciness in the present sample set.

This study provides one of the first quantitative demonstrations, based on trained descriptive sensory analysis, that an image‐based macrostructural descriptor is highly predictive of perceived fibrousness in PBMAs. By integrating image‐based macrostructural analysis with directional compression testing and sensory evaluation, the present work establishes a clear structure–mechanics–perception framework for fibrous plant‐based meat analogues. This framework enables instrumental measurements of fibrous structure to be interpreted in a sensory‐relevant context and provides practical guidance for the development and evaluation of fibrous textures in PBMAs.

## 6 Practical Applications

This study provides practical guidance for the design and evaluation of fibrous textures in plant‐based meat analogues. The findings demonstrate that macrostructural fiber alignment, quantified through image‐based analysis, is strongly linked to perceived fibrousness during consumption. This suggests that rapid imaging tools such as Fiberlyzer can be applied in industrial product development as screening methods to optimize texture before costly sensory trials. In addition, directional mechanical testing can support formulation and processing decisions by providing insight into fracture behavior and structural integrity during mastication. Together, these approaches enable more targeted development of whole‐cut plant‐based products with improved meat‐like bite and expected consumer acceptance. The results are particularly relevant for food manufacturers aiming to enhance sensory quality, accelerate innovation cycles, and reduce reliance on iterative trial‐and‐error product optimisation.

## Author Contributions

**Christina J. Birke Rune:** conceptualization, methodology, investigation, formal analysis, writing – original draft, visualization. **Mathias P. Clausen:** conceptualization, methodology, supervision, funding acquisition, writing – review and editing. **Miek Schlangen:** conceptualization, methodology, investigation, formal analysis, funding acquisition, writing – review and editing, resources. **Davide Giacalone:** conceptualization, methodology, supervision, writing – review and editing, funding acquisition.

## Funding

The work presented in this article is supported by Novo Nordisk Foundation grant NNF23OC0085919. Author C.J.B.R. further acknowledges support from the SDU Climate Cluster through the project “Sustainable dairy replacing food structures”.

## Ethics Statement

Ethical approval for this study was obtained from the University of Southern Denmark's Research Ethics Committee (Approval ID REC520).

## Consent

Written informed consent was obtained from all study participants.

## Conflicts of Interest

The authors declare no conflicts of interest.

## Supporting information


**Appendix S1:** Overview of literature on sensory evaluation of fibrousness in plant‐based meat analogues.
**Table S1:** Classification of studies identified in the literature screening that evaluated fibrousness in plant‐based meat analogues (PBMAs), including sensory methodology and attribute definition.
**Appendix S2:** Representative Images for Fiberlyzer.
**Figure S1:** Three representative photographs per sample used for Fiberlyzer analysis. Owing to within‐sample structural variation, the images are intended as representative examples rather than the complete set of analyzed images.
**Appendix S3:** Calibration of Fiberlyzer. For each image, the Fiber Score was computed using the Fiberlyzer method at different combinations of segmentation threshold % and minimal area %. On a sample level, the computed Fiber Score at each combination of segmentation threshold % and minimal area % were correlated to the expert panel visible fibrousness (Figure S2A). The highest correlation of determination (*R* ^2^) was used as a criteria for parameter selection of threshold and minimal area in the Fiberlyzer method, which was at a threshold of 72% and a minimal fiber area of 0.01% (Figure S2B). These settings were used to compute the Fiber Score of the entire dataset of images in this study.
**Figure S2:** (A) Parameter grid search correlation and (B) corresponding scatter plot of expert panel score versus Fiberlyzer fiber score with a threshold percentile of 72% and a minimal area of 0.01%.
**Appendix S4:** Stress–strain curves of samples.
**Figure S3:** Representative stress–strain curves of sample 1–5 for (A) parallel to fiber direction and (B) perpendicular to fiber direction.
**Appendix S5:** Panel Performance: Main and Interaction Effects.
**Table S2:** Three‐way ANOVA results showing the effects of sample, assessor, replicate (rep), and their interactions for each sensory attribute. Bold values indicate significant effects (*p* < 0.05).

## Data Availability

Data will be made available on request.
