# Supplementary material for: Linking Structural and Mechanical Properties to Multimodal Sensory Perception of Fibrousness in Plant‐Based Meat Analogs
Source: J Texture Stud. 2026 Aug 20;57(4):e70111. doi: 10.1111/jtxs.70111 (PMC13493321; doi:10.1111/jtxs.70111)
Supplement: Supplementary file 1 [file JTXS-57-e70111-s001.docx]

**Supplementary Appendices:**

**Supplementary Appendix S1 – Overview of literature on sensory evaluation of fibrousness in plant-based meat analogues**

A literature search was conducted in Scopus and Web of Science to identify studies evaluating fibrous texture in plant-based meat analogues (PBMAs) using sensory or consumer methods. The primary search was performed on 7 July 2025 and was limited to English-language journal articles published between 2021 and 2026. This search formed part of a broader literature review and therefore employed relatively broad search terms. After applying document type filters and removing duplicates, 679 records remained.

A follow-up search was conducted on 3 January 2026 to identify articles published since the initial search, yielding an additional 137 non-duplicate records. In total, 816 records were screened at the abstract level.

Abstract screening focused on identifying studies that included sensory evaluation, consumer research, and fibrous or fiber-related texture attributes in the context of PBMAs. This resulted in 104 articles being selected for full-text assessment. In addition, two further articles were identified through other sources, giving a total of 106 full-text articles evaluated.

Of the 106 full-text articles assessed, 46 articles did not include any sensory evaluation, 39 articles did not address fibrousness or fiber-related texture attributes, and four articles were review papers. Two articles reported sensory or consumer outcomes but did not provide sufficient methodological detail to allow interpretation, and one article could not be accessed. In many of the excluded studies, texture was addressed only at a general level, typically referring to “texture”, “mouthfeel”, or “structure”, and only occasionally including more specific attributes such as hardness, chewiness, juiciness, or cohesiveness.

This screening process resulted in 14 studies that included sensory evaluation of fibrousness in PBMAs (Table S1). Among these, four studies applied consumer-based methods, including hedonic liking tests and attribute selection approaches such as Check-All-That-Apply (CATA) or Rate-All-That-Apply (RATA) (Auer et al., 2025; Ettinger et al., 2022; Lu et al., 2025; Richter et al., 2024). In these consumer studies, fibrousness was generally not evaluated as a clearly defined sensory attribute, and the studies did not distinguish between visible and oral fibrousness.

The remaining ten studies applied trained sensory panels using descriptive analysis (DA) to evaluate fibrousness in PBMAs. Among these, three studies assessed fibrousness exclusively as visible fibrousness (Fu et al., 2023; Nisov et al., 2024; Zhao et al., 2024), while the remaining seven studies evaluated fibrousness as an oral texture attribute (Barnés-Calle et al., 2024; Farrokhi & Azizi, 2025; Ge et al., 2025; Lee et al., 2024; Rune et al., 2026; Shahbazi et al., 2022; Xue et al., 2025)et al., 2022; Xue et al., 2025).

Among the ten descriptive analysis (DA) studies, six explicitly defined how fibrousness was evaluated (Barnés-Calle et al., 2024; Lee et al., 2024; Nisov et al., 2024; Rune et al., 2026; Shahbazi et al., 2022; Xue et al., 2025). Together with the definition provided in Nisov et al. (2024), fibrousness is described in broadly similar perceptual terms, generally referring to the perception of elongated, filamentous, or strand-like structures during mastication.

Only three studies attempted to relate sensory fibrousness to instrumental measurements (Barnés-Calle et al., 2024; Lee et al., 2024; Shahbazi et al., 2022). In these studies, the instrumental measurements relied primarily on general mechanical texture metrics, such as shear force or texture profile analysis, rather than explicit measures of fiber alignment or directional mechanical anisotropy.

Taken together, these studies demonstrate that fibrousness is recognized as a relevant texture attribute in PBMAs, but they also highlight substantial methodological heterogeneity in how fibrousness is evaluated and interpreted. Differences in evaluation approach, attribute definition, and sensory context limit cross-study comparability and complicate the establishment of quantitative relationships between perceived fibrousness and underlying structural or mechanical features.

Consequently, current literature provides only fragmented insight into fibrousness as a distinct perceptual experience during eating. While instrumental approaches effectively capture macrostructural fiber alignment and mechanical anisotropy, it remains unclear how controlled structural variation translates into oral fibrousness perception, whether instrumental descriptors adequately capture this perception, and how visual access to structural cues may influence texture evaluation during eating.

***Table S1:*** *Classification of studies identified in the literature screening that evaluated fibrousness in plant-based meat analogues (PBMAs), including sensory methodology and attribute definition.*

| **Study** | **Method ^§^** | **Visible fibrousness** | **Oral fibrousness** | **Definition provided** | **Instrumental link** |
| --- | --- | --- | --- | --- | --- |
| Barnés-Calle et al., 2024 | DA | No | Yes | Yes | Yes |
| Farrokhi & Azizi, 2025 | DA | No | Yes | No | No |
| Fu et al., 2023 | DA | yes | No | No | No |
| Ge et al., 2025 | DA | No | Yes | No | No |
| Lee et al., 2024 | DA | No | Yes | Yes | Yes |
| Nisov et al., 2024 | DA | yes | No | yes | No |
| Rune et al., 2026 | DA | No | Yes | yes | No |
| Shahbazi et al., 2022 | DA | No | Yes | yes | Yes |
| Xue et al., 2025 | DA | No | Yes | yes | No |
| Zhao et al., 2024 | DA | yes | No |  | No |
| Auer et al., 2025 | Hedonic liking + RATA | Not specified | Not specified | No | No |
| Ettinger et al., 2022 | Hedonic liking + CATA | Not specified | Not specified | No | No |
| Lu et al., 2025 | Hedonic liking | Not specified | Not specified | No | No |
| Richter et al., 2024 | RATA | Not specified | Not specified | No | No |
| ^§^ DA = Descriptive analysis, CATA = Check-all-that-apply, RATA = Rate-all-that-apply | | | | | |

**Supplementary Appendix S2 – Representative Images for Fiberlyzer**

A minimum of six photographs was acquired and analysed for each sample as described in Section 2.2. Three representative photographs per sample are shown below to illustrate the images used for Fiberlyzer analysis (Figure S1). Owing to within-sample structural variation, these images are intended as representative examples rather than the complete set of analysed images.

|  | **Example 1** | **Example 2** | **Example 3** |
| --- | --- | --- | --- |
| **Sample 1** |  |  |  |
| **Sample 2** |  |  |  |
| **Sample 3** |  |  |  |
| **Sample 4** |  |  |  |
| **Sample 5** |  |  |  |

*Figure S1: Three representative photographs per sample used for Fiberlyzer analysis. Owing to within-sample structural variation, the images are intended as representative examples rather than the complete set of analysed images.*

**Supplementary Appendix S3 - Calibration of Fiberlyzer**

As a different imaging setup, including lighting conditions and camera, were used in this study compared to Ma et al. (2024), a calibration of the Fiberlyzer algorithm was required. A grid search was performed to identify the best threshold and minimal area parameters that aligned with a ground truth determined by the expert panel. The expert panel consisted of 8 participants which also served on the sensory panel. The expert panel was presented with three representative images of each sample in random order and asked to evaluate the visible fibrousness intensity on a 15 cm line scale, anchored from “a little” to “a lot”, as from the DA.

For each image, the Fiber Score was computed using the Fiberlyzer method at different combinations of segmentation threshold % and minimal area %. On a sample level, the computed Fiber Score at each combination of segmentation threshold % and minimal area % were correlated to the expert panel visible fibrousness (Figure S2A). The highest correlation of determination (R^2^) was used as a criteria for parameter selection of threshold and minimal area in the Fiberlyzer method, which was at a threshold of 72% and a minimal fiber area of 0.01% (figure S2B). These settings were used to compute the Fiber Score of the entire dataset of images in this study.

Figure S2: A) Parameter grid search correlation and B) corresponding scatter plot of expert panel score versus Fiberlyzer fibre score with a threshold percentile of 72% and a minimal area of 0.01%.

**Supplementary Appendix S4: Stress-strain curves of samples**

 Directional compression testing using a texture analyser was performed to characterize the mechanical response of the samples. Representative stress–strain curves for compression parallel (Figure S3A) and perpendicular (Figure S3B) to the fibre direction are shown.

Figure S3: Representative stress-strain curves of sample 1-5 for A) parallel to fiber direction and B) perpendicular to fiber direction.

**Supplementary Appendix S5 – Panel Performance: Main and Interaction Effects**

In table S2, the results of the panel performance evaluation are found. For each sensory attribute, three-way ANOVA models were used to test the effects of assessor, replicate, sample, and their interactions. The table presents p-values for the main effects and all two- and three-way interaction terms. Significant effects (p < 0.05) are marked with an asterisk. The sample effect was significant for all evaluated attributes and detailed results pertaining to the samples are given in Table 2 of the paper. In contrast, replicate effects and replicate-related interactions were not significant for any attribute, indicating good repeatability across repeated evaluations. Assessor effects were minor and limited to two attributes, while only one attribute showed a significant sample × judge interaction. No higher-order three-way interactions were observed. Overall, these results confirm a stable and consistent panel performance.

Table S2: *Three-way ANOVA results showing the effects of sample, assessor, replicate (rep), and their interactions for each sensory attribute. Bold values indicate significant effects (p < 0.05).*

| Attribute | Sample | Assessor | Rep | Sample X assessor | Sample X rep | Assessor X rep | Sample X assessor X rep |
| --- | --- | --- | --- | --- | --- | --- | --- |
| Chewiness | **<0.001** | 0.175 | 0.959 | **<0.001** | 0.865 | 0.681 | 0.690 |
| Cohesiveness | **<0.001** | 0.080 | 0.781 | 0.708 | 0.151 | 0.984 | 0.965 |
| Denseness | **<0.001** | 0.234 | 0.886 | 0.771 | 0.886 | 0.884 | 0.971 |
| Fibrous_10 | **<0.001** | **0.047** | 0.377 | 0.444 | 0.531 | 0.763 | 0.831 |
| Fibrous_5 | **<0.001** | 0.086 | 0.456 | 0.704 | 0.614 | 0.847 | 0.782 |
| Hardness | **<0.001** | 0.083 | 0.968 | 0.658 | 0.584 | 0.884 | 0.766 |
| Juicy | **<0.001** | **0.006** | 0.389 | 0.606 | 0.567 | 0.667 | 0.451 |
| Springiness | **<0.001** | 0.121 | 0.986 | 0.745 | 0.613 | 0.907 | 0.831 |
